# Age-Dependent Lipid–Cardiovascular Interplay in Patients at High and Very High Cardiovascular Risk

**DOI:** 10.3390/jcm15062192

**Published:** 2026-03-13

**Authors:** Mirela Baba, Mihaela Ioana Maris, Simina Mariana Moroz, Cristina Gug, Adina Bucur, Constantin Tudor Luca, Ioana Mozos

**Affiliations:** 1 Doctoral School, “Victor Babeş” University of Medicine and Pharmacy, 300041 Timisoara, Romaniasimina.moroz@umft.ro (S.M.M.); 2 Center for Translational Research and Systems Medicine, “Victor Babeş” University of Medicine and Pharmacy, 300173 Timisoara, Romania; 3 Department of Functional Sciences-Pathophysiology, “Victor Babeş” University of Medicine and Pharmacy, 300173 Timisoara, Romania; 4 Center for Advanced Research in Cardiovascular Pathology and Hemostaseology, “Victor Babeş” University of Medicine and Pharmacy, 300041 Timisoara, Romania; 5 Department of Microscopic Morphology, Discipline of Genetics, Faculty of Medicine, “Victor Babeş” University of Medicine and Pharmacy, 300041 Timisoara, Romania; 6 Department of Functional Sciences, Discipline of Public Health, “Victor Babeş” University of Medicine and Pharmacy, Eftimie Murgu Square Nr. 2, 300041 Timisoara, Romania; 7 Department of Cardiology, “Victor Babeş” University of Medicine and Pharmacy, 300041 Timisoara, Romania; 8 Department of Cardiology, Institute of Cardiovascular Diseases, 300310 Timișoara, Romania

**Keywords:** pulse wave analysis, pulse wave velocity, triglycerides, dyslipidemia, triglyceride-glucose index (TyG), lipid balance index, cardiovascular risk, blood pressure, age, early vascular aging

## Abstract

**Objectives:** This study aimed to investigate the associations between serum lipid biomarkers and pulse wave analysis (PWA) variables in patients at high and very high cardiovascular risk, with particular emphasis on age-related differences. **Methods:** Seventy-six patients at high or very high cardiovascular risk were enrolled and stratified into middle-aged (Group 1) and elderly (Group 2). All participants underwent PWA, and multiple serum lipid biomarkers were measured, including composite lipid indices. **Results:** In both age groups, PWA parameters showed significant correlations with serum lipid biomarkers. Systolic blood pressure (SBP) was an independent determinant of the lipid balance index (LBI), while pulse wave velocity (PWV) and SBP were independent determinants of the triglyceride–glucose (TyG) index. PWV correlated with age in both groups and was higher in Group 2 for comparable blood pressure values. In middle-aged patients, diastolic blood pressure (DBP) showed significant, independent associations with triglycerides and TyG, indicating a close link between peripheral vascular resistance and metabolic dysfunction in earlier stages of cardiovascular risk. In elderly patients, SBP and pulse pressure were predominantly associated with lipid-derived indices, reflecting the increasing contribution of large-artery stiffness and lipid-driven vascular remodeling with advancing age. Systematic Coronary Risk Estimation 2 (SCORE2) correlated significantly with PWV, the lipid index (LI), and the LBI. **Conclusions:** Serum lipid biomarkers and PWA-derived hemodynamic variables exhibit a significant, age-dependent interplay in patients with high and very high cardiovascular risk. These findings underscore the importance of age-specific evaluation of lipid–hemodynamic interactions to improve early identification and targeted management of high-risk individuals.

## 1. Introduction

Atherosclerotic cardiovascular disease is still the foremost cause of death worldwide, and arterial hypertension and dyslipidemia represent two of the most common modifiable risk factors driving this burden [1]. Contemporary ESC/ESH guidance emphasizes that blood pressure-related risk is continuous and should be interpreted in the context of total cardiovascular risk, including metabolic risk modifiers such as dysglycemia, obesity, and atherogenic lipid abnormalities [2,3,4]. Accordingly, integrating hemodynamic and metabolic information may refine cardiovascular prevention beyond the isolated evaluation of single risk factors.

Blood pressure is a heterogeneous phenotype with components reflecting distinct underlying mechanisms across the lifespan. With advancing age, progressive arterial stiffening becomes a dominant determinant of systolic blood pressure (SBP) and pulse pressure (PP), driven by reduced arterial compliance and impaired wave reflection [5]. Pulse wave velocity (PWV) is an established indicator of arterial stiffness and vascular aging, supported by reference standards and strong prognostic links to cardiovascular events and mortality [6,7]. In contrast, diastolic blood pressure (DBP) more closely reflects peripheral vascular resistance and neurohumoral regulation, particularly in younger individuals, and remains clinically relevant for cardiovascular risk evaluation and coronary perfusion [8].

Metabolic disturbances, especially insulin resistance and atherogenic dyslipidemia, are closely linked to the development of vascular dysfunction. Triglyceride-rich lipoproteins and lipid-derived indices capture aspects of residual atherogenic risk that may not be fully reflected by isolated lipid fractions [9]. The triglyceride–glucose (TyG) index is a straightforward surrogate marker of insulin resistance and is linked to unfavorable cardiometabolic profiles and vascular dysfunction across both epidemiological and clinical studies [10,11]. Insulin resistance may contribute to impaired vasodilation, sympathetic activation, and sodium retention, thereby influencing blood pressure regulation and vascular remodeling [12,13,14].

Growing evidence indicates that lipid abnormalities are not only structural drivers of atherosclerosis but also active modulators of arterial stiffness. Population-based studies have demonstrated that elevated triglycerides, non-HDL cholesterol, and atherogenic lipid ratios are independently associated with increased pulse wave velocity and augmentation index, even after adjustment for traditional cardiovascular risk factors. In large cohort analyses, dyslipidemia has been linked to accelerated vascular aging and altered wave reflection patterns, suggesting a direct interaction between lipid metabolism and arterial wall mechanics [7,15].

Mechanistic studies further support these clinical observations. Lipid accumulation within the arterial wall promotes oxidative stress, endothelial dysfunction, and low-grade inflammation, leading to collagen deposition and elastin fragmentation- a hallmark of arterial stiffening. Experimental data indicate that triglyceride-rich lipoproteins and remnant particles impair nitric oxide bioavailability and increase vascular smooth muscle tone, thereby influencing both systolic load and pulsatile hemodynamics [16,17,18]. These pathways provide a biological framework linking dyslipidemia to pulse wave analysis-derived indices beyond simple plaque formation.

Despite increasing recognition of metabolic–vascular interactions, the age-specific relationships between serum lipid biomarkers, metabolic indices, and pulse wave analysis–derived parameters remain incompletely characterized. Clarifying these associations may improve the understanding of cardiovascular risk heterogeneity and support more individualized preventive strategies.

Accordingly, the present study aimed to explore the associations between serum lipid biomarkers, lipid- and metabolism-derived indices, and pulse wave analysis parameters in patients with cardiovascular risk factors, with a particular focus on potential age-related differences.

## 2. Materials and Methods

### 2.1. Study Population

Patients with one or more conventional cardiovascular risk factors were consecutively referred for primary cardiovascular disease prevention screening by family physicians from western Romania to the Institute of Cardiovascular Diseases Timișoara. Conventional cardiovascular risk factors considered for inclusion were arterial hypertension, dyslipidemia, diabetes mellitus or prediabetes, obesity (defined as a BMI ≥ 30 kg/m^2^), active smoking, and metabolic syndrome, as defined according to contemporary European guidelines. As detailed in our previous study [19], all participants underwent pulse wave analysis using an oscillometric device, and multiple serum lipid biomarkers were assessed and derived. Additional data on laboratory tests, diagnoses, and treatments were obtained from medical records.

Inclusion criteria: Individuals exhibiting at least one conventional cardiovascular risk factor were eligible for enrollment. All patients had either high risk or very high risk, as determined by the Systematic Coronary Risk Estimation 2 (SCORE2) or the Systematic Coronary Risk Estimation 2-Older Persons (SCOR2-OP).

Excluded patients included those who met the following criteria:

- Had no conventional cardiovascular risk factors or had a low-to-moderate SCORE2 or SCORE2-OP risk;
- Had a documented history of established cardiovascular disease (myocardial infarction or stroke);
- Had acute inflammatory conditions, active malignancy, advanced hepatic disease, or advanced renal failure.

After applying these criteria, the remaining patients were categorized into two groups: those younger than 65 years and those aged 65 years or older.

Patients receiving lipid-lowering therapy were not excluded, as the aim was to reflect real-world high-risk clinical practice. Lipid-lowering treatment was accounted for in the statistical analyses and incorporated into the lipid balance index (LBI).

Subsequently, patients were stratified into two groups: those younger than 65 years and those aged ≥ 65 years. Comparative analyses were conducted between the two groups, and additional correlation analyses were performed within this stratified cohort.

Ethics Approvals: All procedures were conducted in accordance with the principles of the Declaration of Helsinki and were approved by the Ethics Committee for Scientific Research of the “Victor Babes” University of Medicine and Pharmacy (approval No. 24/28 March 2022). Written informed consent was obtained from all participants after providing detailed information about the study objectives, procedures, and potential implications.

### 2.2. Pulse Wave Analysis

The methodologies for pulse wave analysis and assessment of PWV using the Mobil-O-Graph device (IEM GmbH, Stolberg, Germany) were described and published in our previous studies [19,20,21]. Early vascular aging (EVA) was considered in patients with a vascular age exceeding the chronological age [22].

### 2.3. Serum Lipids

Standard serum lipoproteins were measured using a Siemens Dimension clinical chemistry analyzer (Siemens Healthcare Diagnostics Inc., Erlangen, Germany). All blood samples were obtained in the morning after at least 8 h of fasting. The analyzed parameters included total cholesterol (TC), low-density lipoprotein cholesterol (LDL), high-density lipoprotein cholesterol (HDL), non-HDL cholesterol (non-HDL), and triglycerides (TG). Non-HDL cholesterol was derived as TC minus HDL-C. The calculated lipid ratios, representing the overall lipid balance, comprised the Castelli Risk Index I and II (CRI I and CRI II), the lipid index (LI), and the lipid balance index (LBI), as previously described [19]. The LBI is a composite dyslipidemia metric including triglycerides, LDL-C, HDL-C, and lipid-lowering therapy, designed to reflect the overall atherogenic lipid milieu [19].

### 2.4. Metabolic Syndrome (MetS)

Metabolic syndrome was characterized by the presence of obesity together with at least two of the following abnormalities: elevated blood pressure, impaired glucose regulation, and increased non–high-density lipoprotein (non-HDL) cholesterol, indicative of atherogenic dyslipidemia [23].

### 2.5. TyG Index

Insulin resistance (IR) was assessed using the triglyceride–glucose (TyG) index, calculated as ln [triglycerides (mg/dL) × fasting blood glucose (mg/dL)]/2, according to the method originally proposed by Simental-Mendía [24]. A cut-off value > 4.5 was considered indicative of insulin resistance, consistent with prior validation studies [10,25]. The TyG index has been suggested as a simple, low-cost surrogate indicator of insulin resistance in both epidemiological and clinical research [10,26,27].

### 2.6. SCORE2, SCORE2-OP

Systematic Coronary Risk Estimation 2 (SCORE2) and Systematic Coronary Risk Estimation 2–Older Persons (SCORE2-OP) charts were applied to all patients to estimate the risk of fatal and non-fatal cardiovascular events (myocardial infarction, stroke), based on systolic blood pressure, non-HDL cholesterol, sex, age, and smoking status [28].

### 2.7. Statistical Analysis

Statistical analyses and the sample size calculations were performed using MedCalc^®^ Statistical Software, version 23.1.5 (MedCalc Software Ltd., Ostend, Belgium; https://www.medcalc.org; accessed 4 July 2025). Sample size estimation was performed for the correlation analysis, which served as the primary statistical framework of the study. Assuming a moderate expected correlation coefficient (r = 0.30–0.35), with a two-sided alpha level of 0.05 and statistical power of 80%, the minimum required sample size was estimated at 67–73 participants. The final sample of 76 patients, therefore, ensured adequate statistical power to detect clinically relevant associations between lipid biomarkers and pulse wave analysis parameters. The methods applied included Bravais–Pearson and Spearman rank correlation coefficients, partial correlation, comparison of correlation coefficients, chi-square tests for comparison of proportions, independent-samples *t* tests, and regression analysis. Statistical significance was set at *p* < 0.05. Variable normality was assessed using the Shapiro–Wilk test.

## 3. Results

### 3.1. Characteristics of the Patients Included in the Study

The main characteristics of the 76 patients enrolled in the study are included in Table 1.

### 3.2. Correlations

**Figure 1:**
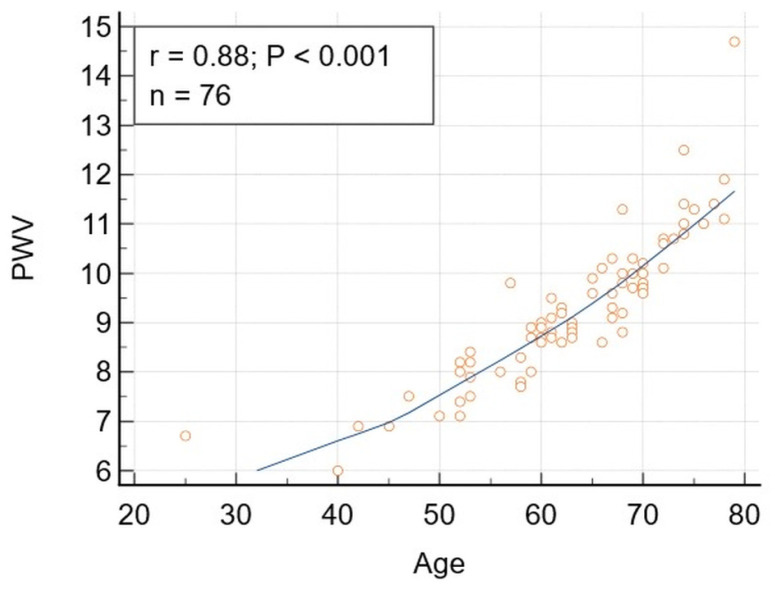
The correlation between pulse wave velocity (PWV) and chronological age (age). The correlation between pulse wave velocity (PWV) (m/s) and chronological age (age) (years): r = 0.88, *p* < 0.001, 95% confidence interval for r: 0.8162 to 0.9223. The age range in the study population was 25–64 years.

Significant correlations were obtained between DBP and TG, and TyG, respectively (Table 2). The significance was not lost after adjusting for lipid-lowering drugs or diabetes mellitus and prediabetes. SBP was also significantly correlated with LBI and TyG, respectively. The correlation between SBP and TyG remained significant after adjusting for LLD, but it lost its significance after adjusting for diabetes mellitus and prediabetes. The only PWA variable significantly correlated with age was PWV (Figure 1). SBP was significantly correlated with LBI (r = 0.24, *p* = 0.034), but this correlation lost significance after adjusting for DM and PD.

Rank correlations were significant for EVA-TyG, and MAP-TG and TyG, respectively (Table 2).

### 3.3. Multiple Regression Analysis

SBP was revealed as an independent determinant of LBI, after adjusting for DBP, AI, and PWV. The relationship remained significant after adjusting for age and BMI. On the other hand, TyG and LBI were found to be independent determinants of SBP. The statistical significance disappeared after adjusting for BMI and for TyG, and the confidence interval became too large for LBI. PWV and SBP were significant determinants of TyG, even after adjusting for AI, DBP, PP, MAP, age, BMI, and LLD (Table 3). TyG was a significant determinant of DBP, after adjusting for TG, BMI, LLD, DM, and PD. SCORE2/SCORE2-OP and LBI were significant determinants of PWV.

### 3.4. Differences Between Patients Considering Age

Next, the patients were divided into two groups, based on age. Group 1 included patients younger than 65 years, while Group 2, included those aged 65 years or older. Significant differences were noticed for age, BMI, PWV, and TyG between Groups 1 and 2 (Table 4). It can be observed that for matched blood pressure variables, PWV was higher in the elderly patients’ group, whereas BMI and TyG were higher in the younger patients’ group. SCORE2 was 14.63 ± 6.96 in Group 1, and 30.61 ± 12.79 in Group 2 (SCORE2 and SCORE2-OP) (*p* < 0.0001).

### 3.5. Correlations in Group 1 (Younger than 65, Sample Size = 40)

**Figure 2:**
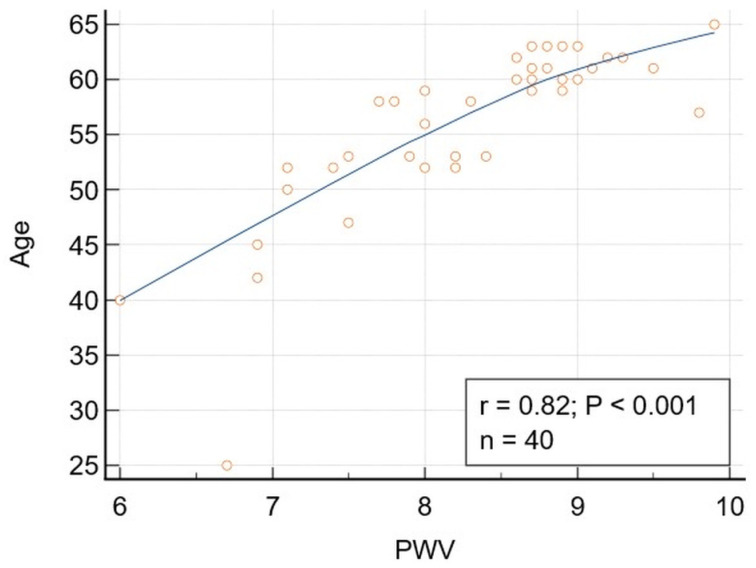
Correlation between Age and PWV in patients younger than 65. Correlation between Age and PWV: r = 0.82, *p* < 0.01, 95% Confidence interval for r: 0.6836 to 0.9015. The correlation remained significant after adjusting for BMI and LLD (r = 0.821, *p* < 0.0001), diabetes mellitus and prediabetes (r = 0.818, *p* < 0.0001). The age range in the study population was 25–64 years.

Significant correlations were obtained for Group 1 patients between DBP and TG and TyG, respectively, and between age and PWV (Figure 2). The correlation between DBP and TG and TyG, respectively, remained significant after adjusting for BMI, PWV, LLD, and diabetes mellitus and prediabetes. Comparing the correlation coefficients, no significant differences were observed between the whole study population and the younger patients’ subgroup when considering the correlation DBP-TG (*p* = 0.542) or DBP-TyG (*p* = 0.819). Despite a higher correlation coefficient for the relationship PWV-Age for the whole study population (0.88) compared to the younger patients’ group (0.82), the differences were not statistically significant (*p* = 0.278).

The correlation between DBP and TG (95% confidence interval for r: 0.08141 to 0.6206) remained significant after adjusting for age, BMI, PWV, and LLD (r = 0.413, *p* = 0.0123) and after adjusting for diabetes mellitus and prediabetes (r = 0.3447, *p* = 0.0317). The correlation between DBP and TyG (95% confidence interval for r 0.07549 to 0.6170) remained significant after adjusting for age, BMI, PWV, and LLD (r = 0.398, *p* = 0.0162) and for diabetes mellitus and prediabetes (r = 0.324, *p* = 0.0443).

**Figure 3:**
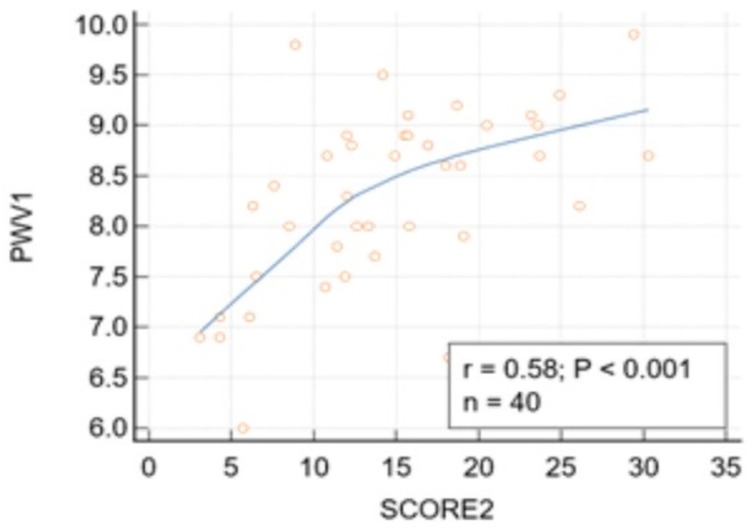
The correlation between PWV and SCORE2 risk in patients younger than 65. Correlation between PWV (m/s) and SCORE2 risk (%) (r = 0.58, *p* < 0.001). 95% Confidence interval for r: 0.3253 to 0.7539.

PWV was significantly correlated with SCORE2 risk (r = 0.58, *p* < 0.001) (Figure 3). The correlation between SCORE2 risk and PWV in patients younger than 65 remained significant after adjustment for age, SBP, and BMI (r = 0.57, *p* = 0.0006). After adjusting for diabetes, prediabetes, and smoking, the correlation also remained significant (r = 55, *p* = 0.0005).

**Figure 4:**
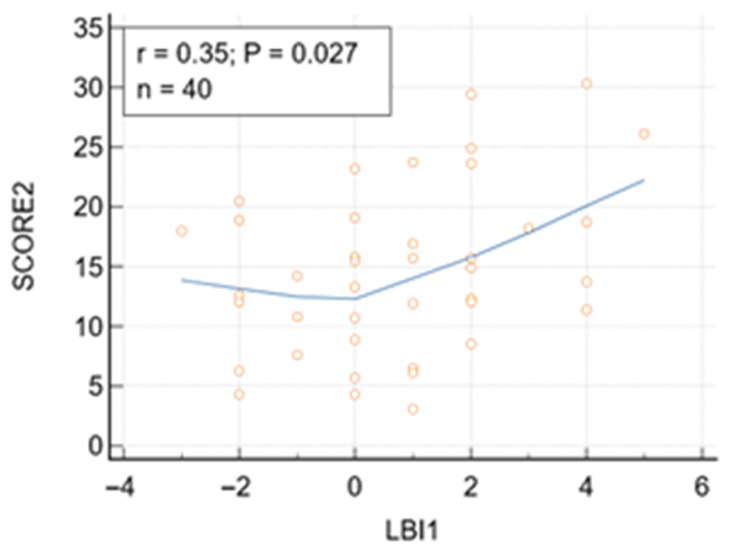
Correlation between lipid balance index (LBI) and SCORE2 in patients younger than 65. Correlation between LBI and SCORE2 (%) risk (r = 0.35, *p* < 0.027). 95% Confidence interval for r: 0.04397 to 0.5970.

Both TG and the TyG index were significantly associated with SCORE2 risk, with positive correlations observed for TG (r = 0.406, *p* = 0.009) and TyG (r = 0.47, *p* = 0.002). Significant correlations were also noticed between SCORE2 and LI and LBI, respectively (Table 5, Figure 4). No additional serum lipid or lipid indices were significantly associated with SCORE2 in patients younger than 65 years.

### 3.6. Correlations in Group 2 (Older than 65, Sample Size = 36)

**Figure 5:**
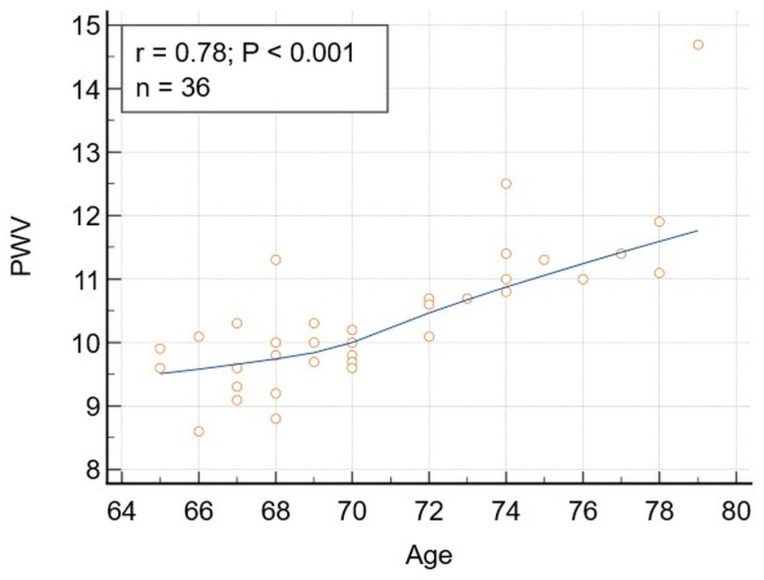
Correlation between Age and PWV in patients older than 65. Correlation between chronological age (years) and pulse wave velocity (PWV) (m/s). 95% Confidence interval for r: 0.6116 to 0.8840. The correlation remained significant after adjusting for BMI (r = 0.7806, *p* < 0.0001) and diabetes mellitus and prediabetes (r = 0.792, *p* < 0.0001).

A significant correlation was obtained in the elderly patients’ group between PWV and age (Figure 5), even after adjusting for BMI, diabetes mellitus, and prediabetes. Despite a lower value of the correlation coefficient (r = 0.78), compared to the values obtained for the whole study population and the younger patients’ group (r = 0.88 and 0.82, respectively), the differences were not statistically significant (*p* = 0.115 and 0.642, respectively).

PWV was strongly associated with estimated cardiovascular risk SCORE2 or SCORE2-OP (r = 0.8, *p* < 0.001) (Table 6). After adjustment for age, the relationship remained significant (r = 0.64, *p* < 0.0001), indicating that the relationship between arterial stiffness and cardiovascular risk was largely age-independent in this cohort. Next, the relation PWV SCORE2/SCORE2-OP was adjusted for SBP and BMI, and statistical significance was preserved (r = 0.86, *p* < 0.001), and was preserved also after adjusting for diabetes, prediabetes (r = 0.73, *p* < 0.0001), and smoking (r = 0.76, *p* < 0.0001).

Besides PWV, PP also significantly correlated with age in group 2 (r = 0.34). However, the correlation lost its significance after adjusting for BMI (r = 0.312, *p* = 0.068), diabetes mellitus, and prediabetes (r = 0.277, *p* = 0.107).

The correlation between LI and SBP was also significant (r = 0.42, *p* = 0.011). The correlation remained significant (r = 0.374, *p* = 0.0317) after adjusting for LLD, age, and BMI, but lost its significance after adjusting for diabetes mellitus and prediabetes (r = 0.268, *p* = 0.119). A positive correlation was found between PP and LI (r = 0.4, *p* = 0.016), which remained significant (r = 0.368, *p* = 0.0349) after adjusting for LLD, age and BMI, but lost its significance after adjusting for diabetes mellitus and prediabetes (r = 0.233, *p* = 0.178). SBP was positively correlated with LBI (r = 0.42, *p* = 0.011). The correlation remained significant (r = 0.398, *p* = 0.0198) after adjusting for age and BMI. Despite higher correlation coefficient compared to the one obtained for the whole population studied, the differences were not statistically significant (*p* = 0.333).

DBP significantly correlated with the LBI, but the correlation lost significance after adjusting for age and BMI (r = 0.297, *p* = 0.0877). PP was also significantly correlated with the LBI, and the correlation remained significant (r = 0.3749, *p* = 0.0289) after adjusting for age and BMI.

SCORE2/SCORE2 OP was significantly correlated with TyG, independent of age and BMI (r = 0.82, *p* < 0.001). SCORE2/SCORE 2OP was positively associated with CRI (r = 0.37, *p* = 0.02) and with CRII (r = 0.43, *p* = 0.007). Rank correlations revealed a significant correlation between PP-TG (rS = 0.409, *p* = 0.013) and SBP-TG (rS = 0.35, *p* = 0.039) (Table 7).

## 4. Discussion

### 4.1. PWV, SCORE2, and Age

The current study identified significant relationships between pulse wave analysis parameters and serum lipid biomarkers in middle-aged and older adults with cardiovascular risk factors. For matched blood pressure variables, PWV was higher in the elderly patient group, illustrating the importance of age. PWV is the only PWA variable correlated with age. In our study, PWV showed a strong positive association with chronological age, consistent with its role as a marker of vascular aging. PWV correlated with age in both young and elderly patients, with no statistically significant differences in the correlation coefficients (*p* = 0.642).

A large body of epidemiological and reference-value research demonstrates that arterial stiffness, as quantified by carotid–femoral PWV (cfPWV), increases progressively with advancing age in healthy populations [29,30]. Notably, the Reference Values for Arterial Stiffness Collaboration pooled over 10,000 subjects and established age-stratified normal values for cfPWV, showing a clear upward trend across age decades that is independent of other cardiovascular risk factors and accentuated at higher blood pressure levels [6]. Similarly, in a well-characterized cohort of 780 healthy individuals aged 10–98 years, PWV increased linearly with age (r^2^ ≈ 0.61; *p* < 0.05), with steeper increases noticed after 50 years [31].

Brachial–ankle PWV (baPWV), a widely used systemic stiffness index, similarly demonstrates a strong age-dependent increase in community and clinical samples, with both cross-sectional and longitudinal analyses reporting progressively higher baPWV values in older age groups and steepest increases observed in late adulthood [32,33]. Reference value studies have established normal baPWV ranges stratified by age and blood pressure, further reinforcing age as a primary determinant of this PWV metric [7].

Moreover, PWV estimated by automated oscillometric techniques (oPWV), including cuff-based measures analogous to ambulatory or office oscillometric devices, also shows a very strong correlation with age (e.g., r ≈ 0.90, R^2^ ≈ 0.81), indicating that oscillometric PWV estimates are highly age-dependent in the adult population [34,35].

This consistent age-related pattern observed across central, peripheral, and oscillometric PWV assessments supports the concept that arterial stiffening is a universal hallmark of vascular aging. It is mechanistically driven by structural alterations of the arterial wall, including elastin degradation and increased collagen deposition. Furthermore, these findings emphasize the value of PWV not only for cardiovascular risk stratification but also as a comprehensive surrogate marker of biological vascular age [36,37].

Pulse wave velocity (PWV) showed a strong association with SCORE2-estimated cardiovascular risk and, importantly, remained an independent predictor after adjustment for age. This finding suggests that PWV captures aspects of vascular risk that are not fully explained by chronological aging alone. While age is a dominant component of SCORE2 calculation, the loss of its independent association after adjustment indicates that vascular stiffness may represent a more direct marker of biological vascular aging than chronological age itself. Previous large cohort studies have demonstrated that PWV provides incremental prognostic information beyond traditional risk factors and improves cardiovascular risk prediction [38,39]. Strömberg et al. previously revealed a clear association between increased cardiovascular risk, as assessed by SCORE2, and arterial stiffness and subclinical coronary atherosclerosis in a general population without diabetes or established cardiovascular disease [40]. The present study included patients with high and very high cardiovascular risk and demonstrated a significant correlation between PWV and SCORE2 and SCORE2-OP, respectively, a correlation that remained significant after adjusting for diabetes, prediabetes, and smoking. SCORE2/SCORE2-OP was found to be an independent determinant of PWV, together with LBI, which refines the lipid/metabolic component, emphasizing the importance of lipid–hemodynamic interactions, besides age and blood pressure, the dominant, near-universal determinants of PWV. The present study is the first mentioning the PWV-SCORE2-LBI crosstalk, consistent with a continuum from risk burden, dyslipidemia, arterial stiffening.

Our results are consistent with the concept that arterial stiffness integrates cumulative exposure to hemodynamic and metabolic stressors and may therefore serve as a functional biomarker of total cardiovascular burden. However, given the cross-sectional design, the present data demonstrate association rather than temporal causation. Prospective studies are required to reveal whether PWV-guided risk stratification improves SCORE2-based prevention strategies.

### 4.2. Diastolic Blood Pressure, Triglycerides, and Triglycerides-Glucose Index

Diastolic blood pressure is an important determinant of coronary perfusion, associated with subclinical myocardial damage and coronary events [8]. Large observational studies have demonstrated a J-shaped relationship between DBP and cardiovascular out comes, with lower DBP values associated with subclinical myocardial damage, reflected by elevated high-sensitivity cardiac troponin levels, and an increased risk of coronary events [8,41,42]. These findings suggest that overly aggressive blood pressure reduction may promote myocardial ischemia through impaired diastolic perfusion. Conversely, elevated DBP has also been associated with adverse cardiovascular outcomes, particularly in younger individuals and those with increased peripheral vascular resistance, where sustained high DBP contributes to left ventricular hypertrophy, microvascular dysfunction, and accelerated atherosclerosis [43,44]. Thus, both low and high DBP appear to confer cardiovascular risk through distinct pathophysiological mechanisms, supporting the concept of an optimal DBP range and the need for individualized blood pressure targets.

The significant correlation of DBP and TyG reveals their potential importance in predicting the risk of coronary events. The observed significant correlation between diastolic blood pressure (DBP), triglyceride (TG) levels, and the triglyceride–glucose (TyG) index highlights a potential mechanistic link between hemodynamic load, insulin resistance, and cardiometabolic risk. Elevated TG and TyG index are well-established surrogate markers of insulin resistance and atherogenic dyslipidemia, which contribute to endothelial dysfunction, increased arterial stiffness, and microvascular impairment [10,12,24,45]. In this context, higher DBP may reflect increased peripheral vascular resistance driven by metabolic dysregulation, thereby amplifying coronary risk through combined metabolic and vascular pathways [43]. Conversely, insulin resistance-related endothelial dysfunction may further exacerbate abnormalities in diastolic blood pressure regulation. Previous studies have demonstrated that the TyG index is independently associated with coronary artery disease, subclinical atherosclerosis, and adverse cardiovascular events, even after adjustment for traditional risk factors [11,24]. From a clinical perspective, the combined assessment of DBP with TG or TyG index may offer a simple and cost-effective approach to identifying individuals at higher risk of coronary events who could benefit from earlier lifestyle or pharmacological interventions targeting both blood pressure control and metabolic dysfunction.

In our study, we found significant correlations between DBP and TG, and TyG, respectively. DBP was an independent determinant of TyG, and TyG was found as an independent determinant of DBP, but the correlation was significant only in the younger group of patients. The bidirectional and age-dependent associations observed in our study, in which diastolic blood pressure (DBP) and the triglyceride–glucose (TyG) index emerged as independent determinants of each other, suggest a close interplay between vascular tone and metabolic dysfunction, particularly in younger individuals. Elevated DBP in younger patients is more likely to reflect increased peripheral vascular resistance rather than arterial stiffness, a hemodynamic pattern strongly influenced by insulin resistance and hypertriglyceridemia [12,46]. Insulin resistance, as captured by the TyG index, promotes endothelial dysfunction, sympathetic activation, and sodium retention, all of which may contribute to higher diastolic blood pressure [47]. Conversely, sustained elevations in DBP may exacerbate microvascular dysfunction and impair insulin-mediated glucose uptake, reinforcing metabolic derangements [48]. However, the present analysis cannot determine directionality, and the noticed relationships should be interpreted as statistical associations rather than evidence of causation.

Triglycerides (TG), the triglyceride–glucose (TyG) index, LI and LBI were significantly associated with SCORE2-estimated cardiovascular risk, whereas no other serum lipid fractions or lipid-derived indices showed comparable associations. This pattern suggests that triglyceride-related metabolic burden may capture a component of cardiovascular risk not fully reflected by traditional lipid markers alone. The TyG index has been extensively validated as a measure of insulin resistance and has been associated with unfavorable cardiometabolic outcomes and vascular dysfunction in large-scale epidemiological studies [10,24,27,49,50]. However, the present cross-sectional design demonstrates association rather than causation, and prospective validation is required to determine whether triglyceride-centered metabolic markers improve SCORE2-based risk stratification.

The absence of a significant association in older patients may be explained by age-related increases in arterial stiffness and pulse pressure, which shift the hemodynamic burden toward systolic blood pressure and attenuate the influence of metabolic factors on DBP [44]. These findings support the concept that the metabolic–vascular interaction between DBP and insulin resistance is particularly relevant at earlier stages of cardiovascular risk, when vascular changes are still potentially reversible.

### 4.3. Systolic Blood Pressure, Pulse Pressure, Lipid Index, and Lipid Balance Index

SBP was found to be an independent determinant of LBI, and LBI was an independent determinant of SBP. Still, the correlation between the two variables was significant only in elderly patients. PP was also correlated with LI and LBI in group 2 of elderly individuals.

In elderly patients, systolic blood pressure (SBP) and pulse pressure (PP) are the predominant hemodynamic components associated with cardiovascular risk, largely reflecting age-related arterial stiffening. With advancing age, progressive loss of arterial compliance leads to increased SBP and widened PP, which in turn enhances pulsatile stress on the coronary and cerebral microcirculation [5,51]. Several studies have demonstrated significant associations between adverse lipid profiles—particularly elevated total cholesterol, low-density lipoprotein cholesterol (LDL-C), and triglycerides—and increased arterial stiffness, thereby contributing to higher SBP and PP in older adults [52,53]. Atherogenic dyslipidemia promotes endothelial dysfunction, vascular inflammation, and structural remodeling of large arteries, accelerating pulse wave velocity and augmenting systolic load [7,45]. Consequently, in the elderly, the correlation between serum lipids and SBP or PP may reflect shared pathophysiological pathways linking lipid-driven atherosclerosis to large-artery dysfunction rather than peripheral resistance–mediated mechanisms typical of younger populations.

Beyond its interaction with systolic load, dyslipidemia may directly contribute to arterial stiffening through several biological mechanisms independent of chronological aging or blood pressure levels. Triglyceride-rich lipoproteins and atherogenic lipid fractions impair endothelial function by reducing nitric oxide bioavailability and increasing oxidative stress [16,17]. This imbalance favors vascular smooth muscle cell activation and enhances vasoconstrictive tone. Chronic exposure to atherogenic lipoproteins promotes low-grade vascular inflammation and extracellular matrix remodeling, characterized by increased collagen deposition and elastin fragmentation—hallmark structural processes underlying arterial stiffening [15,16].

Moreover, lipid accumulation within the arterial wall may accelerate medial calcification and induce phenotypic switching of vascular smooth muscle cells toward a synthetic, pro-fibrotic state, thereby increasing arterial wall rigidity and pulse wave velocity [7,15]. In this context, the association between LBI and PWV observed in our cohort may reflect the cumulative effect of atherogenic lipid burden on vascular structure rather than solely hemodynamic load.

These observations underscore the importance of integrated management of dyslipidemia and systolic hypertension in older patients to mitigate vascular aging and reduce cardiovascular risk. Although dyslipidemia has been associated with endothelial dysfunction and vascular inflammation [16,17], our data reflect shared cardiometabolic burden rather than direct proof of lipid-mediated arterial remodeling.

### 4.4. Strengths and Limitations of the Study

Multiple studies showed that triglycerides, non-HDL cholesterol, and composite lipid indices (e.g., lipid balance index, TyG) are associated with higher PWV and early vascular aging in hypertensive or high-risk patients, supporting our correlations between lipid indices, TyG, and PWA variables [19,54]. The present study has several notable strengths. First, it integrates pulse wave analysis-derived hemodynamic parameters with cardiovascular risk estimation and detailed serum lipid profiling and derived lipid and metabolic indices, including the triglyceride–glucose (TyG) index, allowing a comprehensive evaluation of the interaction between vascular function and cardiometabolic risk. This multimodal approach goes beyond conventional blood pressure or lipid measurements and provides insight into early vascular aging and metabolic–vascular crosstalk. LBI, a multi-component lipid metric, reflects the quality of lipid control and coexisting metabolic injury, and may capture aspects of atherogenic risk not reflected in any single lipid parameter. In a high-risk context with widespread hypertension and older age, residual variability in PWV may be partly explained by the intensity and complexity of dyslipidemia and its treatment, which LBI is designed to represent. Second, the age-based stratification (<65 vs. ≥65 years) enabled the identification of distinct age-dependent patterns, highlighting differential contributions of diastolic blood pressure, systolic blood pressure, and pulse pressure to lipid and metabolic abnormalities in younger and elderly patients. Third, pulse wave analysis was performed using a standardized oscillometric method under controlled conditions, and the analyses were adjusted for relevant confounders, including body mass index, lipid-lowering therapy, and abnormalities of glucose metabolism, strengthening the robustness and internal validity of the findings.

Given the high prevalence of overweight and obesity in our cohort (83%), the potential confounding role of adiposity deserves careful consideration. However, several key associations persisted after adjustment for BMI in both correlation and multivariable regression analyses. In particular, the relationships between DBP and TG, DBP and TyG, as well as the associations between PWV, SBP, and TyG remained statistically significant after BMI adjustment. Moreover, in the age-stratified analysis, the younger group exhibited significantly higher BMI values compared with the elderly group, whereas PWV was markedly higher in the elderly patients despite comparable blood pressure levels. This pattern argues against obesity alone explaining the observed lipid–hemodynamic interactions. While obesity undoubtedly contributes to cardiometabolic burden, our findings suggest that metabolic dysfunction and arterial stiffness interact beyond the sole effect of general adiposity.

Finally, the inclusion of patients from a real-world primary prevention setting enhances the clinical relevance of the results. Arterial stiffness studies have predominantly been conducted in low-to-moderate risk populations, while our paper included high and very high cardiovascular risk patients.

Several limitations should be acknowledged. The cross-sectional design does not allow causal inference, and the observed associations between blood pressure components, serum lipids, and metabolic indices reflect correlations rather than temporal or mechanistic relationships. The relatively modest sample size, particularly after age stratification, may have limited statistical power to detect weaker associations. Although pulse wave velocity was estimated using an oscillometric device rather than carotid–femoral tonometry, previous validation studies support its reliability for cardiovascular risk stratification [55]. Residual confounding related to unmeasured lifestyle factors, medication dose or duration, and inflammatory biomarkers cannot be excluded. In the present study, we just used SCORE2 and SCORE2-OP as generic CVD risk stratifiers. Although they are not guideline-endorsed as the preferred tool in diabetes, it was applied as a uniform baseline risk metric across our heterogeneous, high- and very high-risk cohort that included patients with and without diabetes. These models are known to under-predict absolute risk in high-risk primary-care and older populations, but retain acceptable discrimination; we therefore used them primarily for risk ranking and subgroup comparisons, not for absolute event-rate estimation.

Another limitation stems from the inclusion of patients treated with lipid-lowering drugs, which could potentially affect the validity of certain findings. Nevertheless, the results remained unchanged after adjustment for lipid-lowering therapy in the multiple regression analysis, and this variable was also incorporated into the LBI. Given that the study population consisted of high and very-high-cardiovascular-risk patients, most participants were receiving lipid-lowering and antihypertensive therapy in accordance with current guidelines. While this reflects real-world clinical practice, treatment intensity and duration may influence both lipid biomarkers and arterial stiffness parameters. Lipid-lowering therapy was accounted for in the statistical analyses and incorporated into the LBI; moreover, the principal associations remained significant after adjustment for LLD. Blood pressure variables and PWV were analyzed based on measured values under treatment, reflecting the integrated hemodynamic state at the time of assessment. Nevertheless, residual confounding related to pharmacological therapy cannot be entirely excluded and should be considered when interpreting the findings.

Another limitation is the absence of lipoprotein(a) [Lp(a)] measurements in our cohort. Lp(a) is an established, largely genetically determined cardiovascular risk factor and a target of emerging therapeutic strategies. However, Lp(a) was not routinely assessed in the primary prevention setting from which our patients were recruited. Since the primary objective of this study was to explore age-dependent metabolic–hemodynamic interactions, with particular emphasis on triglyceride-related markers and insulin resistance (TyG index), the absence of Lp(a) does not directly affect the main mechanistic axis investigated. Future studies integrating Lp(a) with pulse wave analysis parameters may provide additional insight into residual cardiovascular risk and vascular aging.

### 4.5. Clinical Implications

The age-dependent patterns observed suggest that cardiovascular prevention may benefit from age-specific metabolic–hemodynamic assessment.

Although the present study included patients at high and very high cardiovascular risk, the observed metabolic–vascular interactions are grounded in biological mechanisms that are not limited to this population. Triglyceride-related metabolic dysfunction and arterial stiffness represent progressive phenomena that may also operate at earlier stages of cardiovascular risk, albeit with potentially lower magnitude. Therefore, these findings may have relevance for broader populations, particularly in identifying early metabolic–hemodynamic alterations before overt cardiovascular disease develops. However, extrapolation to lower-risk or general populations should be performed with caution and requires confirmation in prospective, population-based studies.

In younger individuals, the coupling between DBP and metabolic markers supports early screening for insulin resistance and aggressive lifestyle intervention.

In elderly patients, the stronger association between lipid indices and systolic or pulsatile load emphasizes integrated management of dyslipidemia and systolic hypertension to reduce vascular stress.

Combining metabolic markers such as the TyG index with pulse wave analysis may help identify high-risk phenotypes not fully captured by traditional risk scores, although prospective validation is required. SCORE2 and SCORE2-OP correlations and associations with PWV and lipid indices lead to the conclusion that integrating PWV into risk models may improve cardiovascular risk prediction in middle-aged and older adults. PWV, global risk scores, and lipid/metabolic indices each describe distinct dimensions of vascular damage. Combining them may better identify high-risk individuals than any single metric.

### 4.6. Future Research Directions

Longitudinal studies are needed to clarify temporal relationships between metabolic dysfunction and arterial stiffness. Interventional trials targeting triglycerides or insulin resistance could help distinguish association from causation by evaluating changes in pulse wave parameters following metabolic therapy.

Mechanistic research integrating vascular imaging and endothelial biomarkers may further refine biological pathways linking dyslipidemia to vascular aging. Large population-based datasets should evaluate whether integrated metabolic–vascular markers improve cardiovascular risk prediction beyond established models.

## 5. Conclusions

The present study demonstrated a significant age-dependent interplay between serum lipid biomarkers, metabolic indices, and pulse wave analysis-derived hemodynamic parameters in patients with high and very high cardiovascular risk, underscoring the central role of blood pressure and arterial stiffness in metabolic dysregulation. Pulse wave velocity emerged as the vascular parameter strongly associated with age, confirming its role as a robust marker of vascular aging. In younger patients, diastolic blood pressure showed significant and independent associations with triglycerides and the triglyceride–glucose index, highlighting a close link between peripheral vascular resistance and metabolic dysfunction at earlier stages of cardiovascular risk. In contrast, in elderly patients, systolic blood pressure and pulse pressure were predominantly associated with lipid-derived indices, reflecting the increasing contribution of large-artery stiffness and lipid-driven vascular remodeling with advancing age.

These findings support the concept that metabolic and hemodynamic determinants of cardiovascular risk differ according to age, with insulin resistance–related mechanisms playing a more prominent role in younger individuals, while arterial stiffness and atherogenic dyslipidemia dominate in older patients. The combined assessment of pulse wave analysis parameters with lipid and metabolic indices, particularly the TyG index, may therefore provide complementary and clinically relevant information for cardiovascular risk stratification in primary prevention settings. Overall, our results emphasize the importance of an integrated, age-tailored metabolic–vascular approach to better characterize vascular aging and guide personalized preventive strategies.

## Figures and Tables

**Table 1:** Patient characteristics of the study population.

Parameters	Intervals
**Cardiovascular risk factors**	
Chronological age	63 ± 10 years
Male patients	40 (53%)
Body mass index (kg/m^2^)	29.89 ± 5
Obesity participants	31 (47%)
Obesity and overweight participants	55 (83%)
Smoking participants	29 (44%)
Dyslipidemia patients	62 (94%)
DM participants	19 (29%)
DM and PD patients	23 (36%)
MS participants	28 (42%)
MS criteria	1.43 ± 1.73
**Pulse wave analysis**	
AI	24.84 ± 16.07%
PWV	9.29 ± 1.46 m/s
SBP	134.63 ± 18.21 mmHg
DBP	84.42 ± 11.03 mmHg
PP	50.21 ± 13.14 mmHg
MAP	106.14 ± 17.31 mmHg
EVA	27 (36%)
**SCORE2/SCORE2-OP (%)**	22.2 ± 12.89
**Serum lipids**	
LI	1.41 ± 1.79
LBI	0.59 ± 1.97
TC (mg/dL)	202.04 ± 54.14
TG (mg/dL)	189.01 ± 205.13
Non-HDL (mg/dL)	151.89 ± 53.43
LDL (mg/dL)	127.73 ± 47.94
HDL (mg/dL)	49.592 ± 13.6775
TyG	4.838 ± 0.3825
CRI I	4.33 ± 1.68
**Serum biochemical parameters**	
Fasting plasma glucose	117 ± 40 mg/dL
HbA1c	6.3 ± 1.1%
Creatinine	0.9 ± 0.3 mg/dL
Uric acid	6.4 ± 1.6 mg/dL
AST	25 ± 10 mg/dL
ALT	27 ± 14 mg/dL
**Therapy**	
ACEI	34 (51.5%)
Angiotensin receptor blockers	18 (27.3%)
CCA	34 (51.5%)
Loop diuretics	6 (9.1%)
Mineralocorticoid receptor antagonists	5 (7.6%)
Thiazide-like diuretics	25 (37.9%)
BB	38 (57.6%)
If channel inhibitors	4 (6.1%)
Potassium channel blockers	4 (6.1%)
CAAH	7 (10.6%)
Nitrates	7 (10.6%)
MAID	5 (7.6%)
Antiplatelet drugs	30 (45.5%)
Anticoagulant drugs	4 (6.1%)
Angiotensin receptor/neprilysin inhibitor	2 (3%)
SGLT2I	2 (3%)
GLP 1A	1 (1.5%)
Statins	45 (68.2%)
SCAI	5 (7.6%)
Fibrates	7 (10.6%)
PUF	2 (3%)
Biguanides	8 (12.1%)
SU	6 (9%)
Insulin	5 (7.6%)

Abbreviations: MS = Metabolic syndrome, AI = Augmentation index, PWV = Pulse wave velocity, SBP = Systolic blood pressure, DBP = Diastolic blood pressure, PP = Pulse pressure, MAP = Mean arterial pressure, EVA = Early vascular aging, HbA1c = Hemoglobin A1c, AST = Aspartate aminotransferase, ALT = Alanine aminotransferase, TC = Total cholesterol, TG = Triglycerides, Non-HDL = Non-high-density lipoproteins, LDL = Low-density lipoproteins, HDL = High-density lipoproteins, TyG = Triglyceride-glucose index, CRI I = Castelli Risk Index I, ACEI = Angiotensin-converting enzyme inhibitors, SGLT2I = Sodium-glucose co-transporter-2 inhibitors, GLP 1A = Glucagon-like peptide 1 agonists, DM = diabetes mellitus, PD = prediabetes, LI = Lipid index, LBI = Lipid balance index, CCA = Calcium channel antagonists, BB = Beta-blockers, PUF = Polyunsaturated fats Omega 3, SU = Sulphonylureas, SCAI = Selective cholesterol-absorption inhibitors, CAAH = Centrally active antihypertensives, MAID = Metabolic anti-ischemic drugs, SCORE2 = Systematic Coronary Risk Estimation 2, SCORE2-OP = Systematic Coronary Risk Estimation 2-Older Persons (SCOR2-OP).

**Table 2:** Associations between pulse wave analysis parameters and lipid biomarkers.

Correlations	r (*p*)
LBI-TyG	0.53 (<0.0001)
LI-TyG	0.62 (<0.0001)
EVA-TG	rS = 0.21 (0.0639)
EVA-TyG	rS = 0.24 (0.0353)
DBP-BMI	0.24 (0.036)
DBP-LBI	0.213 (0.065)
DBP-TG	0.27 (0.016)
DBP-TG adjusted for LLD	0.283 (0.014)
DBP-TG adjusted for DM and PD	0.228 (0.049)
DBP-TyG	0.34 (0.003)
DBP-TyG adjusted for LLD	0.349 (0.002)
DBP-TyG adjusted for DM and PD	0.228 (0.049)
SBP-BMI	0.289 (0.012)
SBP-LBI	0.243 (0.034)
SBP-LBI adjusted for DM and PD	0.002 (0.989)
SBP-LI	0.211 (0.067)
SBP-TyG	0.249 (0.029)
SBP-TyG adjusted for LLD	0.266 (0.021)
SBP-TyG adjusted for DM and PD	0.051 (0.667)
SBP-TG	rS = 0.214 (0.063)
MAP-BMI	rS = 0.276 (0.016)
MAP-TG	rS = 0.228 (0.047)
MAP-TyG	rS = 0.264 (0.021)
PP-BMI	0.198 (0.087)
PWV-Age	0.88 (<0.001)
PWV-Age adjusted for DM and PD	0.88 (<0.0001)
PWV-SCORE2/SCORE2-OP	0.84 (<0.0001)

Abbreviations: LBI = Lipid Balance Index, TyG = Triglyceride-glucose index, LI = Lipid Index, EVA = Early vascular aging, TG = Triglycerides, DBP = Diastolic blood pressure, BMI = Body mass index, LLD = Lipid lowering drugs, DM = Diabetes mellitus, PD = Prediabetes, SBP = Systolic blood pressure, MAP = Mean arterial pressure, PP = Pulse pressure, PWV = Pulse wave velocity, rS = Spearman rank correlations, r = Bravais-Pearson correlation coefficient, SCORE2 = Systematic Coronary Risk Estimation 2, SCORE2-OP = Systematic Coronary Risk Estimation 2-Older Persons (SCOR2-OP).

**Table 3:** Results of multiple regression analysis.

Dependent Variable	Independent Variables	Multiple R	R Square	Adjusted R-Square	F
LBI	SBP Adjusted for DBP, AI and PWV	0.2432	0.05917	0.04646	0.0342
LBI	SBP Adjusted for DBP, AI, PWV, Age and BMI	0.2432	0.05917	0.04646	0.0342
TyG	PWV (*p* = 0.035) SBP (*p* = 0.005) Adjusted for AI, DBP, PP and MAP	0.3433	0.1178	0.09365	0.0103
TyG	PWV (*p* = 0.032) DBP (*p* = 0.042) DM_PD (*p* < 0.0001) Adjusted for SBP	0.556	0.309	0.2804	<0.0001
TyG	PWV (*p* = 0.035) SBP (*p* = 0.0045) Adjusted for Age, BMI and LLD	0.3433	0.1178	0.09365	0.0103
SBP	TyG Adjusted for LBI, LI, TC, TG, LDL, HDL, Non-HDL	0.2493	0.06213	0.04946	0.0299
SBP	BMI Adjusted for TyG, age	0.2887	0.08334	0.07095	0.0114
SBP	LBI Adjusted for TyG	0.2432	0.05917	0.04646	0.0342
SBP	LBI (*p* = 0.0335) BMI (*p* = 0.0114) Adjusted for TyG	0.3725	0.1388	0.1152	0.0043
DBP	TyG Adjusted for TG, BMI, LLD	0.3394	0.1152	0.1032	0.0027
DBP	TyG Adjusted for TG, BMI, LLD, DM_PD	0.3056	0.09336	0.08111	0.0073
PWV	SCORE2/SCORE2-OP (*p* < 0.0001) LBI (*p* = 0.0005)	0.865	0.748	0.742	<0.0001

Abbreviations: LBI = Lipid Balance Index, TyG = Triglyceride-glucose index, LI = Lipid Index, TG = Triglycerides, DBP = Diastolic blood pressure, BMI = Body mass index, LLD = Lipid lowering drugs, DM = Diabetes mellitus, PD = Prediabetes, SBP = Systolic blood pressure, MAP = Mean arterial pressure, PP = Pulse pressure, PWV = Pulse wave velocity, AI = augmentation index, CI = Confidence interval, F = Significance level, R square = Coefficient of determination, SCORE2 = Systematic Coronary Risk Estimation 2, SCORE2-OP = Systematic Coronary Risk Esti-mation 2-Older Persons (SCOR2-OP).

**Table 4:** Differences between Group 1 and 2.

Nr.	Variable	Group 1 (*n* = 40)	Group 2 (*n* = 36)	Significance of Differences (*p*)
1	Age (years)	56 ± 8	71 ± 4	<0.0001
2	Sex (male)	22 (55%)	20 (55.56%)	0.961 *
3	BMI (kg/m^2^)	31.64 ± 4.65	27.95 ± 4.95	0.0013
4	PWV (m/s)	8.29 ± 0.89	10.41 ± 1.14	<0.0001
5	AI (%)	26.18 ± 16.09	23.36 ± 16.14	0.45
6	SBP (mmHg)	135 ± 13	135 ± 23	0.99
7	DBP (mmHg)	86 ± 11	83 ± 11	0.104
8	MAP (mmHg)	109 ± 11	103 ± 22	0.199
9	PP (mmHg)	48 ± 11	52 ± 15	0.178
10	EVA	17 (42.5%)	12 (33.33%)	0.414 *
11	TC (mg/dL)	206.35 ± 60.06	197.25 ± 47.09	0.468
12	TG (mg/dL)	223.34 ± 232.77	150.86 ± 164.17	0.125
13	LDL (mg/dL)	128.08 ± 54.30	127.35 ± 40.47	0.948
14	HDL (mg/dL)	47.13 ± 11.85	52.33 ± 15.16	0.098
15	TyG	4.94 ± 0.39	4.72 ± 0.35	0.012
16	CRI I	4.64 ± 1.88	4.01 ± 1.38	0.104
17	Dyslipidemia	36 (90%)	35 (97.22%)	0.208 *
18	LI	1.65 ± 1.79	1.14 ± 1.78	0.216
19	LBI	0.73 ± 1.97	0.44 ± 1.98	0.986
20	LLD	0.93 ± 0.79	0.69 ± 0.52	0.145
21	Diabetes mellitus and prediabetes	13 (32.5%)	10 (27.78%)	0.657 *

* *p* was obtained by chi-squared test, comparison of proportions. The other *p*-values were obtained by an independent samples *t*-test. Abbreviations: BMI = Body mass index, PWV = Pulse wave velocity, AI = Augmentation index, SBP = Systolic blood pressure, DBP = Diastolic blood pressure, MAP = Mean arterial pressure, PP = Pulse pressure, EVA = Early vascular aging, TC = Total cholesterol, TG = Triglycerides, LDL = Low-density lipoproteins, HDL = High-density lipoproteins, TyG = Triglyceride-glucose index, CRI I = Castelli Risk Index I, LI = Lipid Index, LBI = Lipid Balance Index.

**Table 5:** Correlations in Group 1.

Nr.	Correlation Between	r (*p*)
1.	MAP-TyG	0.311 (0.051)
2	MAP-TG	0.300 (0.06)
3	DBP-TG	0.38 (0.015)
4	DBP-TyG	0.38 (0.016)
5	PWV-Age	0.82 (<0.001)
6	PWW-SCORE2	0.58 (*p* < 0.001)
7	SCORE2-TG	0.40 (*p* = 0.09)
8	SCORE2-TyG	0.47 (*p* = 0.02)
9	SCORE2-LBI	0.35 (*p* = 0.027)
10	SCORE2-LI	0.46 (*p* = 0.002)

Abbreviations: r = Bravais-Pearson correlation coefficient, MAP = Mean arterial pressure, TyG = Triglyceride-glucose index, TG = Triglycerides, DBP = Diastolic blood pressure, PWV = Pulse wave velocity, SCORE2 = Systematic Coronary Risk Evaluation 2.

**Table 6:** Correlations in Group 2.

Nr.	Correlation Between	r (*p*)
1	PWV-Age	0.78 (<0.001)
2	PP-Age	0.34 (0.041)
3	SBP-LI	0.42 (0.011)
4	PP-LI	0.40 (0.016)
5	SBP-LBI	0.42 (0.011)
6	DBP-LBI	0.33 (0.050)
7	PP-LBI	0.39 (0.018)
8	PWW- SCORE2/SCORE2 OP	0.8 (<0.001)
9	TyG-SCORE2/SCORE2 OP	0.48 (0.003)
10	CRI-SCORE2/SCORE2 OP	0.37 (0.02)
11	CRII-SCORE2/SCORE2 OP	0.43 (0.007)
12	SCORE2/SCORE2-OP-LI	0.45 (0.006)
13	SCORE2/SCORE2-OP-LBI	0.47 (0.004)

Abbreviations: r = Bravais-Pearson correlation coefficient, PWV = Pulse wave velocity, PP = Pulse pressure, SBP = Systolic blood pressure, LI = Lipid Index, LBI = Lipid Balance Index, DBP = Diastolic blood pressure, SCORE2 = Systematic Coronary Risk Evaluation 2, SCORE2-OP = Systematic Coronary Risk Evaluation 2 Older Persons, TyG = Triglyceride-glucose index, CRI = Casteli Risk Index I, CRII = Casteli Risk Index II.

**Table 7:** Rank correlations in Group 2.

Nr.	Correlation Between	rS (*p*)
1	SBP-TG	0.35 (0.039)
2	PP-TG	0.409 (0.013)

Abbreviations: rS = Spearman rank correlations, SBP = Systolic blood pressure, TG = Triglycerides, PP = Pulse pressure.

## Data Availability

The data presented in this study are available on request from the corresponding authors.

## Abbreviations

The following abbreviations are used in this manuscript:

ACEI	Angiotensin-Converting Enzyme Inhibitors
AI	Augmentation Index
ALT	Alanine Aminotransferase
ARB	Angiotensin receptor blockers
AST	Aspartate Aminotransferase
baPWV	Brachial–Ankle Pulse Wave Velocity
BB	Beta- blockers
BMI	Body Mass Index
CAAH	Centrally active antihypertensives
CCA	Calcium channel antagonists
cfPWV	Carotid–Femoral Pulse Wave Velocity
CRI I	Castelli Risk Index I
CVD	Cardiovascular disorders
DBP	Diastolic Blood Pressure
DM	Diabetes Mellitus
EVA	Early Vascular Aging
GLP 1A	Glucagon-Like Peptide 1 Agonist
HbA1c	Glycated Hemoglobin
HDL	High-Density Lipoprotein Cholesterol
IR	Insulin resistance
LBI	Lipid Balance Index
LDL	Low-Density Lipoprotein Cholesterol
LI	Lipid Index
LLD	Lipid-Lowering Drugs
MAID	Metabolic Anti-Ischemic Drugs
MAP	Mean Arterial Pressure
MS	Metabolic Syndrome
non-HDL	Non-HDL Cholesterol
oPWV	PWV Estimated by Automated Oscillometric Techniques
PD	Prediabetes
PP	Pulse Pressure
PUF	Polyunsaturated fats
PWA	Pulse Wave Analysis
PWV	Pulse Wave Velocity
r	Bravais-Pearson Correlation Coefficient
rS	Spearman Rank Correlations
SCORE2	Systemic Coronary Risk Estimation 2
SCORE2-OP	Systematic Coronary Risk Estimation 2-Older Persons
SGLT2I	Sodium-Glucose Co-Transporter-2 Inhibitors
SBP	Systolic Blood Pressure
SCAI	Selective Cholesterol- Absorption Inhibitors
SU	Sulphonylureas
TC	Total Cholesterol
TG	Triglycerides
TyG	Triglyceride-Glucose Index
